# Effect of microvesicles from *Moringa oleifera* containing miRNA on proliferation and apoptosis in tumor cell lines

**DOI:** 10.1038/s41420-020-0271-6

**Published:** 2020-06-04

**Authors:** Marina Potestà, Valentina Roglia, Marialaura Fanelli, Elisa Pietrobono, Angelo Gismondi, Simone Vumbaca, Rick Gildas Nguedia Tsangueu, Antonella Canini, Vittorio Colizzi, Sandro Grelli, Antonella Minutolo, Carla Montesano

**Affiliations:** 1 Department of Biology, University of Rome ‘Tor Vergata’, via della Ricerca Scientifica 1, 00133 Rome, Italy; 2 Department of Experimental Medicine and Surgery, University of Rome “Tor Vergata”, Via Montpellier 1, 00133 Rome, Italy

**Keywords:** Apoptosis, Drug development

## Abstract

Human microvesicles are key mediators of cell–cell communication. Exosomes function as microRNA transporters, playing a crucial role in physiological and pathological processes. Plant microvesicles (MVs) display similar features to mammalian exosomes, and these MVs might enhance plant microRNA delivery in mammals. Considering that plant microRNAs have been newly identified as bioactive constituents in medicinal plants, and that their potential role as regulators in mammals has been underlined, in this study, we characterized MVs purified from *Moringa oleifera* seeds aqueous extract (MOES MVs) and used flow cytometry methods to quantify the ability to deliver their content to host cells. The microRNAs present in MOES MVs were characterized, and through a bioinformatic analysis, specific human apoptosis-related target genes of plant miRNAs were identified. In tumor cell lines, MOES MVs treatment reduced viability, increased apoptosis levels associated with a decrease in B-cell lymphoma 2 protein expression and reduced mitochondrial membrane potential. Interestingly, the effects observed with MOES MVs treatment were comparable to those observed with MOES treatment and transfection with the pool of small RNAs isolated from MOES, used as a control. These results highlight the role of microRNAs transported by MOES MVs as natural bioactive plant compounds that counteract tumorigenesis.

## Introduction

Cells are in constant communication with each other. This communication is mainly mediated by extracellular vesicles, which are small transporters made up of a lipidic membrane that act as carriers of molecules^1^. In this context, extracellular vesicles have recently been suggested to be able to transfer their cargo into exogenous recipient cells, mediating communication between unrelated species^1–5^.

Plant microvesicles (MVs) are a heterogeneous class of vesicles that play unique functions, as protective compartments, for intercellular transport of multiple materials, contributing to plant growth and development, defense responses, and plant–microbe symbiosis^6^.

Plant MVs have been classified as “exosome-like” because their morphology and density are similar to those of mammalian exosomes^7^.

Several in vitro and in vivo studies have suggested that edible plant-derived MVs can accumulate in mammalian cells, and have important functional effects on recipient cells. These plant-derived MVs seem to participate in intestinal tissue homeostasis in healthy subjects and have anti-inflammatory effects in tumor cells^8^.

Small RNA and microRNA (sRNA, miRNA), together with lipids, secondary metabolites, and proteins, have been shown to be present in plant extracellular vesicles^8^.

MVs appear to enhance sRNA stability; in fact, “microvesicles and its content in sRNAs”, have been shown to be resistant to pasteurization, homogenization and ultrasonic treatments^6,9^.

These MVs may mediate plant–animal communication, having a role in cross-kingdom regulation processes, by delivering plant sRNAs and miRNAs to mammalian systems^7,10–13^.

MiRNAs are short stretches of noncoding RNAs that play an essential role in gene regulation; they regulate protein expression at the post-transcriptional level in animals and plants. More than four hundred conserved miRNAs have been identified from plants, and in silico and in vitro functional validation have highlighted the possible role of plant miRNAs as regulators of protein expression in mammals^11,14–21^.

According to these data, sRNAs and miRNAs present in medicinal plants might act as bioactive compounds in mammals.

In many African communities, *Moringa oleifera* Lam. (MO) is widely used for preparations of traditional remedies. The benefits of MO-based preparations are scientifically documented^22,23^. These studies demonstrated that MO bioactivity depends on the presence of different classes of plant secondary metabolites^24,25^. In 2016, the miRNome of MO was sequenced, showing the presence of several conserved miRNAs^26–30^. Potestà et al.^31^ reported that MO seed aqueous extract (MOES), is able to differentially regulate proliferation and apoptosis in healthy and cancer cells and that this ability is associated with the presence of miRNAs.

Therefore, in the current work the MVs present in the MOES previously studied^31^ were extracted and characterized and the ability of these vesicles to enter human tumor cells and induce proapoptotic and antiproliferative effects were investigated.

## Results

### Characterization and delivery of MVs extracted from MOES

Plant Mvs fulfill two roles: miRNA protection and transport of into recipient cells^14,17,32^.

In the present research, size and content of MOES MVs were characterized; moreover, their role in cell host was investigated.

**Fig. 1:**
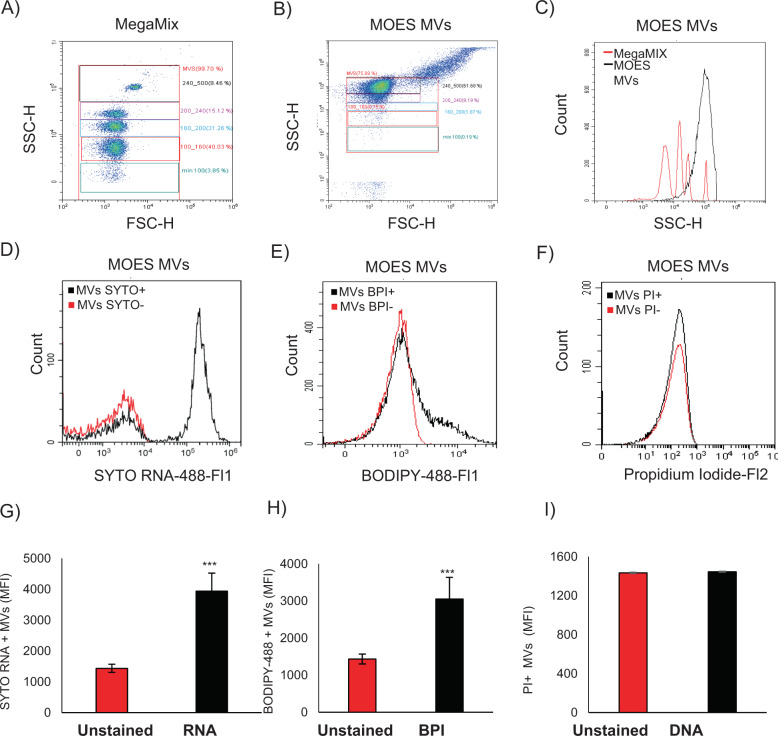
Characterization of MVs extracted from MOES. A representative pseudo-dot plot (FSC-H vs SSC-H, **a**) of the standardized fluorescent (FITC-A) nanosized particles of different sizes (100–160 nm, 160–200 nm, 200–240 nm, 240–500 nm) from the Megamix-Plus SSC kit used as a control for the analysis of the dimensions of the vesicles in the sample. The FSC-H and SSC-H parameters of MOES MVs extracted from 10 mg MOES is shown on **b**. In a representative histogram of the SSC-H parameter, the Megamix reference particles show four peaks corresponding to the dimensions described above (**b**, red line) compared with the control, the MVs extracted from MOES (black line, **c**) show a greater presence of microvesicles with a diameter between 240 and 500 nm. The MVs marked with probes for RNA, lipids, and DNA were analyzed by flow cytometry. On **d**, a representative overlay histogram of the SYTO RNA marked MVs compared with the unmarked MVs. **g** represents the mean ± SD of the SYTO RNA Mean Fluorescence Intensity (MFI) of three independent measurements made using three different samples of MVs. For the analysis of lipid content, a BODIPY probe was used: **e** shows a representative histogram overlay of the BODIPY-positive MVs compared with the unmarked MVs. **h** represents the mean ± SD of the BODIPY MFI of three independent measurements made using three different samples of MVs. For the analysis of DNA content, a propidium iodide (PI) probe was used: **f** shows a representative histogram overlay of the PI-positive MVs compared with the unmarked MVs. **i** represent the mean ± SD of the PI MFI of three independent measurements made using three different samples of MVs. Data are reported as the mean of three different experiments ± SD. *t* test was used; symbols indicate significant differences: ***p < 0.001.

Using the Megamix-Plus SSC (Biocytex, France) standard as a reference in flow cytometry analysis, we identified a population of 100–500 nm MOES MVs (Fig. 1a), as described in the Materials and Methods.

The dimensions of MOES MVs were comparable to those of the Megamix control. Specifically, 59% of the MOES MVs analyzed were between 240 and 500 nm in size, 3.65% were between 200 and 240 nm, and only 2% were < 200 nm (Fig. 1b, c).

The contents of MOES MVs, analyzed by spectrophotometer, showed an absence of DNA (Fig. 1f, i) but presence of RNA, proteins, and lipids: the MOES MVs present in 10 mg of MOES contained 1.63 ± 0.03 µg/µl of protein and 7.9 ± 0.75 ng/µl of RNA (Table 1a).

**Table 1a:** Characterization of MVs derived from 10 mg of MOES.

	Concentration
Total RNA	7.9 ± 0.75 ng/µl
Protein	1.63 ± 0.03 µg/µl
DNA	undetectable

Mean ± SD of three independent measurements. The content of RNA and DNA was dosed using a spectrophotometer (Pharmacia Biotech Ultrospec 3000), for protein quantification BIORAD method was used.

Using specific probes to identify the presence of lipids, RNA and DNA, Flow cytometry analysis was performed. Among the MOES MVs, a significant number of SYTO RNA and BODIPY (BPI) positive MVs (Fig. 1d, e, Table 1b) with a significant increase in Mean Fluorescence Intensity (MFI) (Fig. 1g, h) was observed.

**Table 1b:** Characterization of Lipids, DNA, and RNA contents with BODIPY fluorescent probe (Thermo Fisher Scientific, USA), propidium iodide, and SYTO RNA-staining methods, respectively.

	No. of events	% of positive events
Total events in MVs gate	19224.5 ± 3536	–
BPI + (Lipids)	3844.5 ± 365	20.63 ± 0.58
PI + (DNA)	0	0
SYTO RNA +	7762.65 ± 135	42.49 ± 3.65

Mean ± SD of three independent measurements. Flow cytometry analysis was performed via Cytoflex (Beckman Coulter, USA) and CytExpert 2.2 software (Beckman Coulter, USA).

Table 1b also contains data on the quantification of RNA, DNA, and lipids determined by flow cytometry analysis of a 50 µl MVs sample extracted from 10 mg of MOES. The results are reported as the number of events measured in 50 µl of sample that were positive for the probes used to detect the presence of lipids, DNA, and RNA. As reported in Table 1b, there are a % of 42.49 ± 3.65 positive cells for the RNA probe and 20.63 ± 0.58 for the BPI probe. The characterization of MOES MVs showed that lipids, proteins and RNA are present, whereas DNA was not found.

**Fig. 2:**
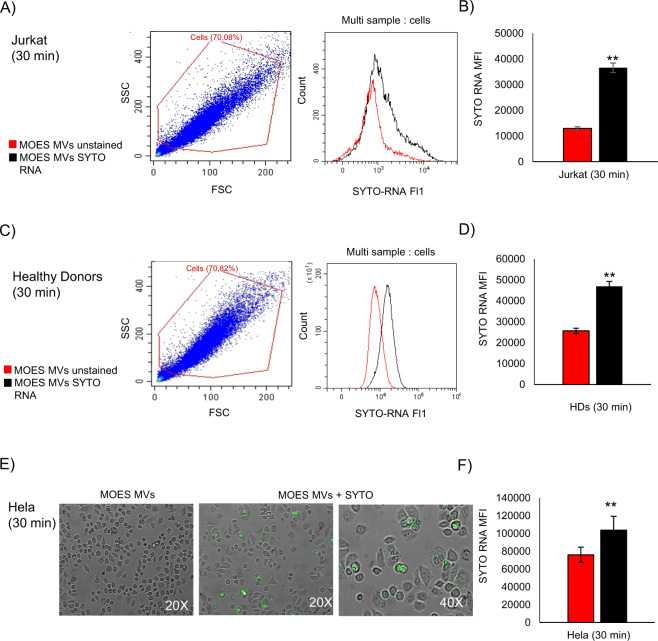
Delivery of MOES MVs to Jurkat cells, HeLa cells, and PBMCs from healthy donors. The presence of stained MOES MVs in Jurkat cells, HeLa cells and PBMCs from healthy donors was investigated after 30 minutes of treatment with MOES MVs stained with SYTO RNA and analyzed by flow cytometry analysis or microscopy. Jurkat and Healthy donors were gated for cells (**a** and **c**, left side). Representative overlay histograms of Jurkat cells (**a**, right side) and PBMCs (**c**, right side) labelled with SYTO RNA-stained MOES MVs (black line) compared with unlabeled cells (red line) are presented. The MFI of SYTO RNA-positive Jurkat cells and PBMCs is reported in **b** and **d**, respectively, representing the mean ± SD of the MFI of three independent measurements made using three different samples of MVs. In HeLa cells, delivery was assessed by fluorescence microscopy analysis of RNA-positive HeLa cells. **e** shows a representative delivery experiment analyzed by fluorescence microscopy: the left picture shows HeLa cells treated only with MVs; the middle and left pictures of **e** shows the cells at two magnifications (×20 and ×40) after incubation for 30 minutes with MVs marked with the RNA probe. The MFI of SYTO RNA-positive HeLa cells is represented by the mean ± SD of three independent measurements of three different samples of MVs **f**. Data are reported as the mean of three different experiments ± SD. *t* test was used; symbols indicate significant differences: **p < 0.001 stained *vs* unstained cells.

Host cell internalization of SYTO RNA-labelled MOES MVs was observed in Jurkat cells and PBMCs from HDs by flow cytometry (Fig. 2a–d) and HeLa cells through microscopy (Fig. 2e, f). In both cell lines and in PBMCs from HDs, a significant increase in fluorescence intensity was observed 30 minutes after treatment (Fig. 2b, d, f), indicating their ability to naturally enter into the cell without needing other carrier.

### Cytotoxic effect of MOES and MOES MVs

To obtain information about the effects of MOES MVs on cell viability, Jurkat and HeLa cells, and PBMCs from HDs were treated with MOES at a concentration ranging from 0 to 50 mg/ml and with the number of MOES MVs purified from each investigated MOES concentration. Seventy-two hours after treatment, cell viability was analyzed using a trypan blue assay.

**Fig. 3:**
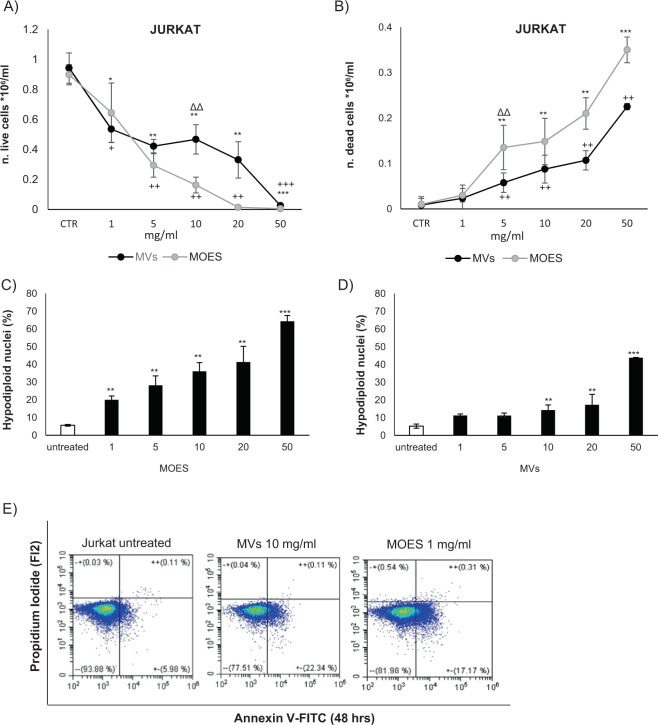
Cytotoxic effect of MOES and MOES MVs on Jurkat cells. Cell viability and mortality analyzed by the Trypan blue exclusion assay in Jurkat cells **a** and **b** after 72 h treatment with MOES at concentrations ranging from 0 to 50 mg/ml or MVs purified from the corresponding concentrations of MOES. Control cells were incubated for the same time with an equivalent volume of distilled water. The results are expressed as number of trypan blue-negative **a** or trypan blue-positive **b** cells. Effects of MOES **c** and MOES MVs **d** on apoptosis in Jurkat cells. Apoptosis was evaluated as the percentage of hypodiploid nuclei by flow cytometry analysis after 72 h of incubation. **e** shows a representative dot plot of the percentages of annexin V-, propidium iodide-, and annexin V/propidium iodide-positive Jurkat cells treated with MOES and MOES MVs after 48 hours of incubation. Data are reported as the mean of three different experiments ± SD. Symbols indicate significant differences in viability/death and apoptosis: *p < 0.05 **p < 0.01, ***p < 0.001 MOES-treated *vs* untreated cells. ^+^*p* < 0.05, ^++^*p* < 0.01, ^+++^*p* < 0.001 MOES MVs-treated *vs* untreated cells. ^Δ^*p* < 0.05, ^ΔΔ^*p* < 0.01, ^ΔΔΔ^*p* < 0.001 comparisons of the concentrations of the different treatments (MOES *vs* MOES MVs) able to significantly modify cell viability or death. *t* test was used for Apoptosis and Trypan blu assay analysis; ANOVA and a Bonferroni multiple comparison test for annexin V/propidium iodide assay were used.

MOES and MOES MVs treatment at 1 mg/ml induced a significant reduction in Jurkat cells viability that was dose dependent (Fig. 3a).

**Fig. 4:**
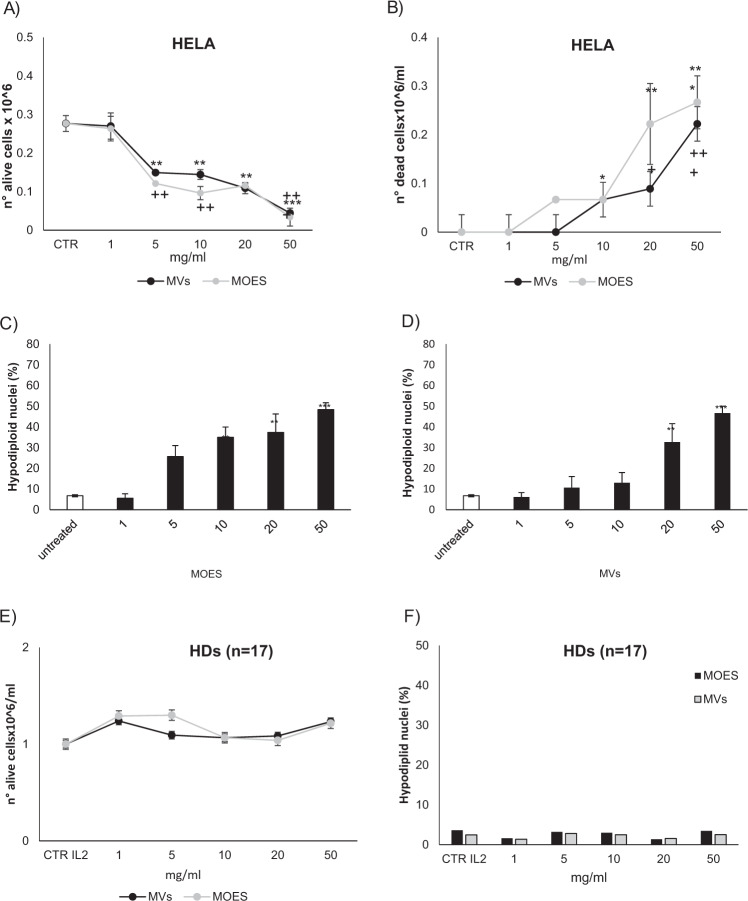
Cytotoxic and apoptotic effects of MOES and MOES MVs on HeLa cells and PBMCs from HDs. Cell viability and mortality analyzed by the Trypan blue exclusion assay in HeLa cells (**a** and **b**) and PBMCs **e** after 72 h treatment with MOES at concentrations ranging from 0 to 50 mg/ml or MOES MVs purified from the corresponding concentrations of MOES. Control cells were incubated for the same time with an equivalent volume of distilled water. The results are expressed as number of Trypan blue-negative **a** and **e** or trypan blue-positive **b** cells. Effects of MOES **c** and MOES MVs **d** on apoptosis in HeLa cells and PBMCs **f**. Apoptosis was evaluated as the percentage of hypodiploid nuclei by flow cytometry analysis after 72 hours of incubation. Data are reported as the mean of three different experiments ± SD. Symbols indicate significant differences in viability/death and apoptosis: *t* test was used for Apoptosis and Trypan blu assay analysis; *p < 0.05, **p < 0.01, ***p < 0.001 MOES-treated *vs* untreated cells. ^+^*p* < 0.05, ^++^*p* < 0.01, ^+++^*p* < 0.001 MOES MVs-treated *vs* untreated cells.

HeLa cells were more resistant to MOES and MOES MVs treatment, showing a significant reduction in viability, starting at 5 mg/ml for both MOES and MOES MVs (Fig. 4a).

The treatments had different cytotoxic effects: both MOES and MOES MVs significantly increased the number of trypan blue-positive Jurkat cells. However, starting at 5 mg/ml, MOES was significantly more toxic than MOES MVs treatment at the same concentrations (Fig. 3b).

In HeLa cells, 10 mg/ml MOES and MOES MVs were required to significantly increase the number of trypan blue-positive cells, confirming that HeLa cells were more resistant to this type of treatment. Moreover, there was no difference in the induction of cell death between MOES and MOES MV-treated HeLa cells, at any of the analyzed concentrations (Fig. 4b).

Considering these results, the concentrations of MOES and MOES MVs able to reduce cell viability to 50% of that at time 0 (EC50) after 72 h of treatment were calculated (Table 2).

**Table 2:** Cytotoxic effect of MOES and MOES MVs treatments on Jurkat and HeLa cell lines and PBMCs from Healthy donors (*n* = 17).

	MOES FW mg/ml	MOES MVs mg/ml
*Jurkat*		
EC50	13.63 ± 0.024^a^	31.57 ± 0.004
LD50	8.42 ± 0.026	22.91 ± 0.011
*HeLa*		
EC50	22.31 ± 0.03^a^	45.63 ± 0.025
LD50	11.63 ± 0.01	12.31 ± 0.01
*HDs*		
EC50	>100	>100
LD50	>100	>100

Mean ± SD of three independent measurements. Trypan blue assay results from the different concentrations of extracts were compared using ANOVA and a Bonferroni significant difference test as a multiple comparison test.

^a^MOES *vs* MOES MVs, *p* < 0.01.

Jurkat cells were more susceptible to MOES treatment than to MOES MVs treatment. Indeed, 13.63 ± 0.024 mg/ml MOES was enough to reduce Jurkat cell viability by 50% (EC50), whereas the number of MOES MVs corresponding to 3.57 ± 0.004 mg/ml MOES was necessary to reduce viability by 50%.

Moreover, MOES induced 50% cell death (LD50) more effectively than MOES MVs. Specifically, the concentration of MOES that induced 50% Jurkat cell death (LD50) was 8.42 ± 0.026 mg/ml, whereas the number of MOES MVs corresponding to 22.91 ± 0.001 mg/ml MOES was necessary to induce 50% cell death (Table 2).

In the adhesive HeLa cell line, treatment with MOES MVs was less toxic than treatment with the MOES preparation, as previously observed in Jurkat cells. Indeed, 22.31 ± 0.03 mg/ml MOES was required to inhibit cell viability by 50%, which is 10 mg more than that in Jurkat cells. MOES MVs were also less toxic for HeLa cells than the Jurkat cells, requiring the number of MOES MVs corresponding to 45.63 ± 0.025 mg/ml MOES to reach a 50% inhibition of cell growth. These data confirmed that adhesive cells exhibited a greater resistance to these treatments than suspension cells and that both cells lines exhibited a greater resistance to MOES MVs than to MOES (Table 2).

Previously, MOES treatment was shown to have these toxic effects on different tumor lines but not on PBMCs from HDs, suggesting that MOES treatment may specifically target cancer cells^31^.

The effects of MOES MVs treatment on cell viability were therefore investigated on PBMCs from HDs stimulated with IL2 (Fig. 4e).

Neither MOES nor MOES MVs treatment affected the viability/death of PBMCs from HDs at any analyzed concentration (Fig. 4e), confirming the ability of these extracts to have toxic effects specifically on cancer cells and not on healthy ones.

### Effect of MOES and MOES MVs on cell cycle and apoptosis

To understand whether the effects on viability/mortality were associated with an induction of apoptosis and cell cycle perturbation, we stained the cells with PI after 72 h of treatment and performed flow cytometry analysis. In Jurkat cells, there was a significant increase in the percentage of apoptotic cells starting at 1 mg/ml MOES (Fig. 3c).

Treatment with higher MOES concentrations resulted in a significant increase in apoptosis in a concentration-dependent manner. Treatment with MOES MVs caused a significant increase in apoptosis starting at 10 mg/ml, proving that this type of treatment is less toxic (Fig. 3d). The cell cycle analysis showed a significant reduction in the number of events and in the percentage of cells in G2 phase related to a significant increase in the cells undergoing to apoptosis (Table S2).

In the HeLa cell line, higher concentrations of MOES and MOES MVs were needed to induce apoptosis than in the Jurkat cell line. Indeed, 5 mg/ml MOES was needed to observe a significant induction of apoptosis (Fig. 4c).

Treatment at higher concentrations increased the percentage of hypodiploid cells in a dose-dependent manner. In addition, MOES MVs were less toxic than MOES, and a concentration of 20 mg/ml was necessary to obtain a significant increase in apoptosis (Fig. 4d). As for the Jurkat, the cell cycle analysis showed a significant reduction of the HeLa cells in phase G2 and an increase in the number and percentage of apoptotic cells (Table S2).

Apoptosis in PBMCs appeared to be comparable to that in the untreated control at all concentrations tested, confirming that only cells of tumor origin were susceptible to the treatments (Fig. 4f).

Considering these data, we chose the following concentrations for subsequent studies: 1 mg/ml MOES and 10 mg/ml MOES MVs for the Jurkat cell line and 5 mg/ml MOES and 20 mg/ml MOES MVs for the HeLa cell line.

To confirm that the treatments induced apoptosis at these concentrations, the Jurkat and HeLa cells were stained early after treatment (48 hours) and assessed using the annexin V/PI assay to distinguish early apoptotic cells (annexin V+/PI−) and late apoptotic/necrotic cells (annexin V+/PI+)^33^. The results shown in Fig. 3e are representative of two experiments performed on Jurkat cells. Table 3 reports the data obtained on Jurkat and HeLa cells.

**Table 3:** Percentage of Annexin V (Anx V), propidium iodide (PI), and annexin V/propidium iodide (Anx V/PI)-positive cells in Jurkat and HeLa cells treated with MOES and MOES MVs after 48 hours of incubation.

Jurkat	Anx V + PI−	Anx V + PI +	Anx V − PI +	Anx V − PI −
Untreated	4.80 ± 2.40	0.18 ± 0.03	0.51 ± 0.20	92.56 ± 0.14
MVs 10 mg/ml	21.20 ± 1.07***	0.77 ± 0.15*	0.81 ± 0.29 ns	88.65 ± 0.36
MOES1 mg/ml	18.92 ± 0.13***	0.25 ± 0.14*	0.08 ± 0.15 ns	82.61 ± 0.24*
*HeLa*	*Anx V* *+* *PI−*	*Anx V* *+* *PI+*	*Anx V* *−* *PI* *+*	*Anx V* *−* *PI−*
Untreated	10.80 ± 2.40	0.16 ± 0. 41	0.52 ± 0.20	89.73 ± 0.14
MVs 20 mg/ml	23.16 ± 1.12**	0.14 ± 0.26	0.18 ± 0.9 ns	83.16 ± 0.61*
MOES 5 mg/ml	21.80 ± 0.74***	0.25 ± 0.10	0.61 ± 0.05 ns	81.71 ± 0.24*

Mean ± SD of two independent measurements, *p < 0.05, **p < 0.01, ***p < 0.001; MOES or MOES MVs vs untreated (ANOVA–Bonferroni multiple comparison test).

After 48 hours of treatment with MOES, Jurkat and HeLa cells showed a significant increase in the percentage of annexin V-positive cells compared with untreated cells. Conversely, the levels of late apoptotic/necrotic cells (annexin V+/PI+) in the sample exposed to the treatment were similar to those detected in the control sample. Treatment with MOES MVs significantly increased annexin V-positive cells in both tumor cell lines.

In addition, in the sample treated with MOES MVs, the levels of late apoptotic/necrotic cells (annexin V+/PI+) were similar to those detected in the control sample. Clearly, these experiments asserted that MOES and MOES MVs were able to specifically induce cell death by apoptosis.

### miRNA expression profile of MOES MVs and MOES

The previous results demonstrated the antiproliferative and proapoptotic effects of MOES on cancer cells. Considering the results published in Potestà et al.^31^, we investigated the possible role of miRNAs carried by MOES MVs in these specific mechanisms.

Starting from the characterization of the *M. oleifera* miRNome^29^, the presence of specific miRNAs belonging to 20 conserved families of plant miRNAs was evaluated in the *mol*-sR pool^31^. Here, by qPCR analysis, we detected and quantified the presence of these miRNAs (miRs) in the pool of sRNAs extracted from the MOES MVs. Among the 21 *mol*-miRs belonging to the 20 most conserved plant miRNA families, 19 were found in MOES MVs. The comparison between the miRNAs present in MOES MVs and those identified in the MOES highlighted that conserved plant miRNAs were present at high concentrations in both samples (Table 4). The amount of miRNAs in MOES and MOES MVs preparations were comparable, except that miR396a and miR396c, which belong to a specific plant miRNA family (miRNA modulators for growth-regulating factors)^34^. In particular, these miRNAs were highly expressed in MOES MVs, compared with MOES (Table 4).

**Table 4:** Characterization of microRNA from MOES and MOES MVs.

	MOES MVs	MOES	MOES MVs *vs* MOES *p* < 0.001
*mol-*miR 159c	417752.12	137806.81	
*mol-*miR 156a	1262.69	763.93	
*mol-*miR 858	30.32	934.01	
*mol-*miR 395a	302.81	267.29	
*mol-*miR 162a	64.10	655.88	
*mol-*miR 171d	1736.89	712.77	
*mol-*miR 2118a	1025.63	447.98	
*mol-*miR 482b	1011.50	341.87	
*mol-*miR 396c	272614606.23	327.94	***
*mol-*miR 396a	49546768.16	10.91	***
*mol-*miR 168a	98.52	71.13	
*mol-*miR 397-5p	80.02	6.26	
*mol-*miR 167-5p	827.31	943.77	
*mol-*miR 166i	561.17	642.38	
*mol-*miR 398b	7.79	3.28	
*mol-*miR 160 h	9.33	4.37	
*mol-*miR 393a	7.12	20.64	
*mol-*miR 159a	57940.62	12096.38	
*mol-*miR 858b	1	1	

Relative RT-qPCR expression analysis of the most conserved *mol*-miRNAs contained in the pool of sRNAs extracted from MOES and MOES MVs. The analysis was carried out in three independent biological experiments and expressed as fold change with respect to *mol*-miR858b expression, previously normalized with a housekeeping gene (5 S rRNA). The relative expression of *mol*-miRs was quantified by the 2^−ΔΔCt^ method and reported as 2^–ΔΔct^ × 10^5^.

### Computational prediction of genes targeted by mol-miRs recovered from MOES and MOES MVs

Following the characterization of the miRs content in MOES MVs, a bioinformatic analysis was performed to identify the possible human genes targeted by the miRs present in the *mol*-sR pool and involved in the apoptosis mechanism^16,20,35^.

The results show that among the 19 most conserved *mol*-miRs found in MOES MVs, 12 could potentially bind human mRNAs involved in apoptosis (Table 5).

**Table 5:** Bioinformatics analysis of possible human mRNA targets of miRs contained in the *mol*-sR pool and involved in the apoptosis process.

miRNA	No. of target	mRNA	No. of target	mRNA
		Antiapoptotic		Proapoptotic
*mol*-mir160h	21	bfar, bnip3l, ripk2, igf1r, cd40lg, bcl-2l10, nol3, bnip3, tnf, bcl-2l2, bcl-2l1, xiap, nfkb1, hrk, akt1, bag3, mcl1, dapk1, naip, birc3, birc5	4	ripk2, tnf, hrk, akt1
*mol*-mir482b	17	mcl1, bfar, igf1r, hrk, braf, bcl-2l2, bcl-2l1, dapk1, akt1, nol3, bcl2, birc3, cd27, il10, xiap, bax, bag3	3	hrk, akt1, bax
*mol*-mir166	14	bax, braf, mcl1, cd27, bnip3, bcl-2l2, bag1, nfkb1, cflar, igf1r, hrk, birc5, bcl2, akt1	2	hrk, akt1
*mol*-mir 159c	9	cd40lg, il10, birc6, naip, dapk1, tnf, bcl-2l10, bcl-2l1, bnip3l	2	tnf, bnip3l
*mol*-mir2118a	5	tnf, bax, akt1, bcl-2l2, bnip3	4	tnf, bax, akt1, bnip3
*mol*-mir167f-3p	5	tnf, akt1, bcl-2l2, cd40lg, bnip3l	3	tnf, akt1, bnip3l
*mol*-mir156e	4	bag1, bag3, cflar, bcl-2l1		
*mol*-mir395d	4	bag1, akt1, il10, bnip3l	2	akt1, bnip3l
*mol*-mir393a	3	bnip2, bnip3l, dapk1	1	bnipl3
*mol*-mir397a	2	dapk1, ripk2	1	ripk2
*mol*-mir858b	1	nol3		
*mol*-mir396a	1	il10		

Among them, four (*mol*-miR160h; *mol*-miR482b; *mol*-miR166; *mol*-miR 159c) showed the possibility to modulate the expression of >10 genes with antiapoptotic activity, which has been shown to be deregulated in tumorigenic mechanisms.

### Effect of miRs contained in MOES MVs on proliferation and apoptosis BCL2 mediated

In previous works, the ability of plant miRNAs^16^ to induce cell death by BCL2-mediated apoptosis in tumor cells has been demonstrated. Moreover, the antiproliferative and proapoptotic properties of MOES were correlated to BCL2 modulation^31^.

For these reasons, MOES MVs treatments were carried out on Jurkat and HeLa cell lines to understand whether the effects of MVs-carried miRs on viability and apoptosis were BCL2-mediated.

Jurkat and HeLa cell lines were treated with the concentrations of MOES MVs and MOES previously chosen. In addition, a purified *mol*-sR pool was used as a positive control in transfection experiments, to determine the ability of the MVs to enter the cell naturally and the efficacy of the treatment directly mediated by the miRs transported by the MVs.

**Fig. 5:**
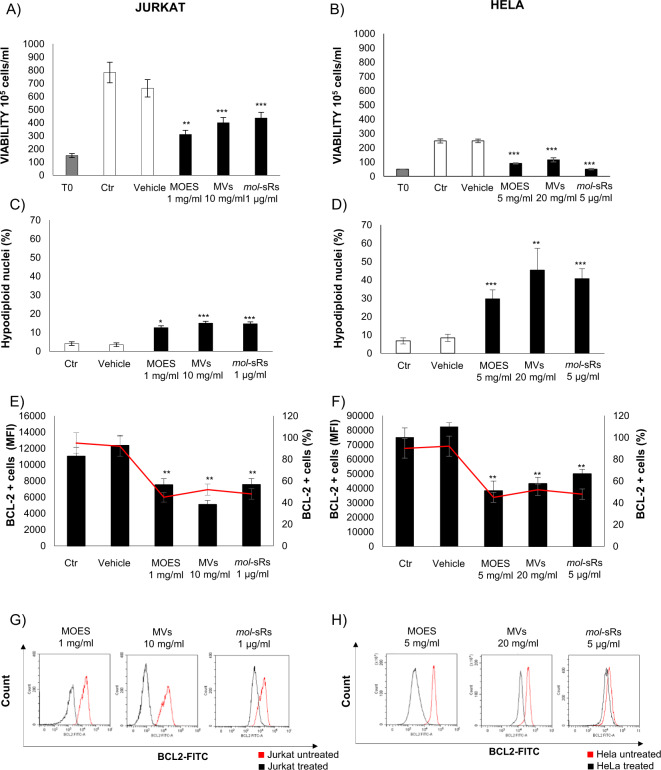
Characterization of the BCL2-mediated antiproliferative and proapoptotic effects of MOES and MOES MVs. **a** and **b** show the viability of Jurkat and HeLa cells, respectively, measured with the Trypan blue exclusion assay. Jurkat cells were treated for 72 hours with 1 mg/ml MOES or MVs corresponding to 10 mg MOES or were transfected with 1 µg/ml *mol*-sRs as a positive control. HeLa cells were treated with 5 mg/ml MOES or MVs corresponding to 20 mg MOES or were transfected with 5 µg/ml *mol*-sRs. The data are reported as the mean ± SD of the number of live cells × 10^6^/ml. Apoptosis was evaluated as the percentage of hypodiploid nuclei by flow cytometry analysis (**c**, **d**) and reported as the percentage of apoptotic cells. BCL2 intracellular protein expression in Jurkat and HeLa cells was evaluated by flow cytometry analysis of live cell gates after 72 hours of treatment. **e**, **f** report the MFI (black barr) and percentage (red line) of BCL2-positive Jurkat and HeLa cells from three independent biological experiments. One representative overlay histogram of BCL2 protein expression in Jurkat **g** and HeLa **h** cells treated with MOES, MVs or *mol*-sRs compared with untreated cells (red line). Data are reported as the mean ± SD of three independent experiments performed. Symbols indicate significant differences: *p < 0.05, **p < 0.01, ***p < 0.001 for all treated *vs* untreated cells. Comparisons between treated and untreated cells were all conducted using *t* test.

After 72 h, compared with the control, all treatments induced a significant reduction of proliferation in both Jurkat and HeLa cells (Fig. 5a, b). Specifically, Jurkat cells showed a significant decrease in the number of alive cells in presence of 1 mg/ml MOES, the number of MVs corresponding to 10 mg/ml (Fig. 5a) and 1 µg/ml *mol*-sRs. A reduction in cell proliferation was evident in HeLa cells treated with 5 mg/ml MOES, the MOES MVs number corresponding to 20 mg/ml MOES and 5 µg/ml *mol*-sRs (Fig. 5b).

Treatment with MOES, MOES MVs, and *mol*-sRs induced a significant increase in apoptosis in both analyzed cell lines. All treatments determined a significative increase in the percentage of hypodiploid nuclei in Jurkat and HeLa cells respect to untreated cells (Fig. 5c, d). The proapoptotic effect was further investigated by evaluating the expression of BCL2 protein through flow cytometry analysis. Treatment with MOES MVs induced a significant decrease in the percentage (red line) and MFI (black barr) in BCL2-positive cells (Fig. 5e, g for Jurkat and F, h for HeLa).

Notably, all the effects of MOES MVs were comparable to those of MOES and *mol*-miR. Taken together, these data suggest that MOES MVs induce a reduction in cell growth and an increase in apoptosis in tumor cell lines that is associated with a significant downregulation of BCL2 protein expression. The transfection of tumor cells with the purified *mol*-sR pool had a comparable effect to MOES and MOES MVs treatments, suggesting that this proapoptotic and response is due to the presence of the miRs in the analyzed extracts.

### Effects of MOES and MOES MVs on mitochondrial membrane potential

Mitochondria play an important role in BCL2-mediated apoptosis: the collapse of the mitochondrial membrane potential (MMP) mirrors the early events leading to apoptotic cell death^36,37^. MMP collapse was detected with the cationic dye JC-1: MMP collapse is associated with an increase in the accumulation of monomeric JC-1, low-red fluorescent forms in the cytoplasm.

**Fig. 6:**
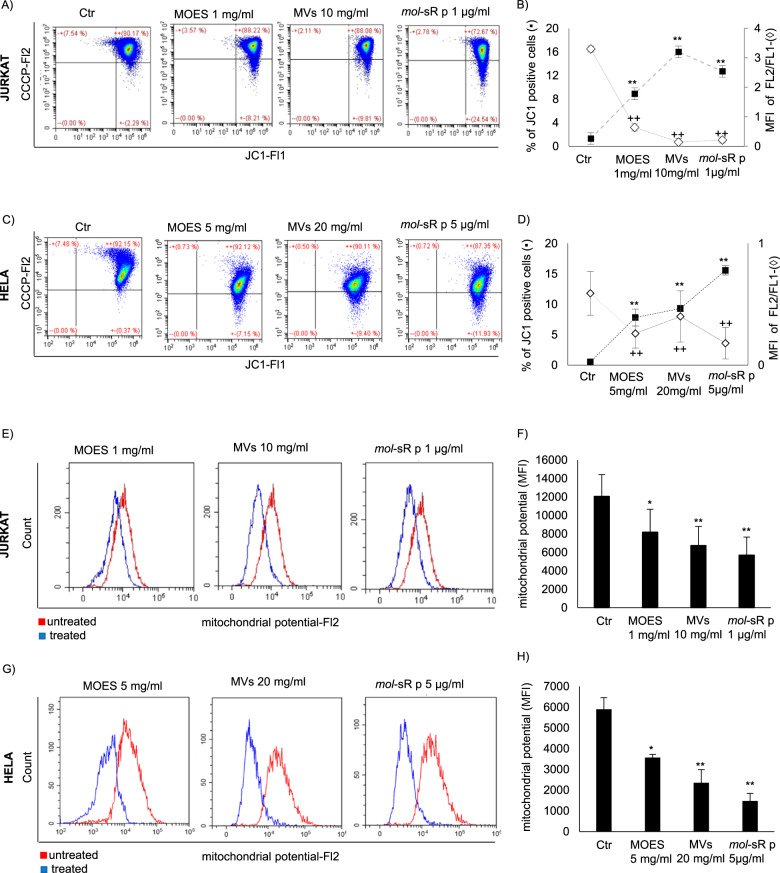
Effects of MOES and MOES MVs on MMP in Jurkat and HeLa cells. The mitochondrial potential in the Jurkat and HeLa cell lines was evaluated by flow cytometry analysis using JC-1 and MitoTracker Red FM probes on live cell gates after 72 h of treatment with MOES, MVs, or *mol*-sRs. **a** and **c** show a representative pseudo-dot plot of JC-1-stained Jurkat and HeLa cells. The percentage of monomeric JC-1-positive cells and the JC-1 red/green mean fluorescence ratios are represented in **b** for Jurkat cells and **d** for HeLa cells. The results of three independent experiments performed for each cell line are expressed as the mean ± SD of the percentage of cells accumulated in the LR quadrant (black square), as well as the red/green mean fluorescence intensity ratio (white rhombus). Representative overlay histograms of MMP expression in Jurkat and HeLa cells stained with MitoTracker Red FM (**e** and **g**) 72 h after treatment compared with MMP expression in untreated cells. The MFI of MitoTracker Red FM-positive cells from three independent biological experiments is reported in **f** and **h**. Data are reported as the mean ± SD of three independent experiments performed. ANOVA and a Bonferroni multiple comparison test were used; symbols indicate significant differences: *p < 0.05, **p < 0.01; all treated *vs* untreated cells.

Thus, the percentage of monomeric JC-1-positive cells in the presence of MOES, MOES MVs and *mol*-sRs was investigated to reflect the number of cells undergoing apoptosis. Cells treated with CCCP exhibited high levels of both red (FL2) and green (FL1) fluorescence, whereas samples treated with MOES, MOES MVs, and *mol*-miRs showed a significant progressive decrease in high-red fluorescent aggregates and an accumulation of cells positive for the monomeric forms of JC-1 in the lower right (LR) quadrant of the cytogram (Fig. 6a, c). At the same time, the percentage of monomeric JC-1-positive cells in the UR+LR quadrant of the cytogram increased in both cell lines with all treatments. Consistent with the MMP collapse induced by the treatments, values of red/green mean fluorescence ratios progressively and significantly decreased following treatment in all cell lines assayed (Fig. 6b, d and Table 6).

**Table 6:** Detection of MMP in Jurkat and HeLa cells.

Jurkat	% of JC-1-positive cells	MFI (FL2/FL1)
CTR	1.3 ± 1.41	3.3 ± 0.75
MOES1 mg/ml	8.9 ± 0.95*	0.64 ± 0.70*
MVS 10 mg/ml	12.70 ± 4.07**	0.21 ± 0.00**
*mol*-sR 1 µg/ml	26.00 ± 2.06**	0.14 ± 0.12**
*HeLa*	*% of JC-1-positive cells*	*MFI (FL2/FL1)*
CTR	0.53 ± 0.10	0.59 ± 0.48
MOES 5 mg/ml	7.82 ± 1.38**	0.26 ± 0.22**
MVS 20 mg/ml	9.31 ± 0.53**	0.40 ± 0.41**
*mol*-sR 5 µg/ml	15.57 ± 0.76**	0.18 ± 0.13**

ANOVA and a Bonferroni multiple comparison test was used. Mean ± SD of three independent measurements. *p < 0.05; **p < 0.01; MOES or MOES MVs *vs* untreated.

Mitochondrial function was also determined by the MitoTracker red assay. During MMP collapse, MitoTracker Red is retained in mitochondria, and the MFI is directly correlated with mitochondrial polarization status. Viable untreated cells were highly fluorescent, whereas in all treatment conditions, there was a significant decrease in fluorescence indicative of apoptotic cells (Fig. 6e–h).

Taken together, these results clearly showed the ability of the *mol*-miRs present in MOES MVs to regulate mitochondria-mediated antiproliferative and proapoptotic mechanisms in cancer cells.

## Discussion

In recent years, several studies have reported beneficial effects of MO on human health^38–40^. The Food and Agriculture Organization of the United Nations (FAO) recommends the consumption of MO, especially for pregnant women, nursing mothers and young children, because it is rich in antioxidants and other essential micronutrients that are lacking in malnourished populations living in underdeveloped and developing countries^41^.

The whole plant is used, from roots to seeds^40^. Literature has reported that MO strengthens and stimulates the immune system owing to the presence of antiradical molecules, minerals, and essential amino acids that are also able to maintain proper blood circulation and fluidization, thereby benefiting the circulatory and cardiovascular system^38,40–44^. MO also has a high anti-inflammatory, which is often at the basis of pathologies such as cancer, cardiovascular diseases, dementia, and arthritis^40,43^.

Zhang^18^ and others works^16,20,45^ introduced the cross-kingdom interaction concept in which miRNAs present in plant extracts, introduced by diet, are able to control gene expression in human cells. Different studies^21,26,46,47^ have confirmed this hypothesis: plant sRNAs containing miRNAs may be considered a new class of micronutrients responsible for the medical properties of plants.

MiRNAs are short nucleotide sequences that interact with mRNA to regulate gene expression at the post-transcriptional level.

How exogenous plant miRNAs ingested with food persist in the mammalian intestine for several hours and are present in body fluids affecting gene expression in humans has been widely discussed^48,49^. Plant miRNAs present a methyl group linked to a 2’ hydroxyl on their 3’ ends that increases their stability during cooking processes, protects them from enzymatic digestion, improve their bioavailability and promotes their regulatory activity in humans^46^. Owing to these characteristics, the use of plant miRNAs in therapeutic practices is of interest. Furthermore, the ability of plant miRNAs to regulate human mRNA stability and translation and their presence in biological fluids has been demonstrated^16,20,35^. What remains to be understood is how these miRNAs can cross the intestinal barrier. Considering the instability of naked RNA, the ability of plant miRNAs to resist pepsin, pancreatin, and bile can be attributed to the presence of protein complexes and lipid vesicles that encapsulate them, protecting them from degradation. The presence of MVs in plants has raised many questions about their biological relevance and potential health benefits. Recent data have demonstrated that MVs from food might have unsuspected functions in mouse digestive tract and thus might participate in whole-body homeostasis^8^. Moreover, in the literature, plant extracellular vesicles, which carry bioactive molecules, such as proteins, mRNAs, and miRNAs, have been shown to act on biological mechanisms in recipient cells. A recent study demonstrated the ability of stained vesicles to cross the intestinal barrier, showing their presence in other regions of the body, such as the liver, spleen, and lymphatic circulation, in mice with colitis^8^.

For several years, we had studied the effects of natural substances, specifically the bioactive component of the seed aqueous extract of *MO* obtained using a traditional African procedure^31^: the antiproliferative and proapoptotic MOES properties on tumor cells were demonstrated, and these effects could be attributed to the presence of miRs contained in the aqueous extract.

In this work, we demonstrated that MOES MVs (carrying miRs) are able to enter human cells and modulate activities related to viability and apoptosis in tumor cell lines.

After extracting MVs from aqueous plant extracts, we characterized the bioactive components (proteins, DNA, RNA, and lipids) of MOES MVs and assessed the dimension of MOES MVs through flow cytometry analysis.

MOES MVs were found to contain proteins, RNA, and lipids but not DNA.

As expected, RNA characterization revealed the presence of miRs belonging to the most conserved plant miRNA family.

Bioinformatic analysis is widely used to evaluate the possible interactions between miRNAs and their mRNA targets^10,14,20,35^. Considering our previous works in which the miRNome of MO^29^ was sequenced and the proapoptotic and antiproliferative effects of MOES were evaluated^31^, here, we identified the possible human genes targeted by plant miRNAs and involved in apoptosis and cell proliferation processes. The obtained results showed the presence of *mol*-miRs, targeting different mRNAs involved in the antiapoptotic mechanisms (Table 5).

Among them, BCL2 transcript was found: BCL2 protein is one of the main factors that regulate tumorigenesis in cell lines of epithelial origin^16,35^ and appear to be the target of both endogenous and exogenous miRNAs^16,35,50,51^. In our previous works, BCL2 protein expression in lymphoid and monocytoid tumor lines was inhibited by treatment with specific plant miRNAs^16^ and MOES^31^. In both studies, the HD cells (PBMCs) were not sensitive to any type of treatment, underlining the ability of MOES and plant sRNAs to have a specific antitumor activity.

Considering these results, the effect of MOES MVs carrying miRs on the viability and apoptosis mechanisms mediated by BCL2 were analyzed.

MOES MVs had lower toxicity respect to MOES. This difference could be owing to the purification process, which allowed the elimination of the most toxic molecules present in MOES (tannins and alkaloids^42,52^).

For this reason, 1 and 5 mg/ml MOES were sufficient in Jurkat and HeLa cells, respectively, to induce a significant increase in apoptosis and a decrease in cell viability. However, to obtain the same results, the number of MOES MVs contained in 10 or 20 mg/ml MOES were needed for Jurkat cells and HeLa cells, respectively.

These modifications in viability and apoptosis were associated with a significant decrease in the expression of BCL2 protein.

The permeabilization of the mitochondrial membrane represents a key event for programmed cell death^31,53–57^. BCL2-mediated all treatments caused a significant reduction in mitochondrial membrane potential associated with apoptosis induction.

The observed results demonstrate that MOES MVs, which carry plant miRNAs, can naturally penetrate inside cells and have proapoptotic effects that are comparable to those of MOES but more specific and less toxic.

Environmental exposure and interactions with genetic factors play an important role in human health. In this study, the epigenetic activity carried out by plant-derived miRNAs contained in MOES MVs was demonstrated to regulate human cellular processes at the post-transcriptional level. Moreover, plant miRNAs have been shown to be fundamental bioactive molecules, suggesting the possible use of plant MVs as a natural vehicle to treat humans, even with plant miRNAs.

## Materials and methods

### MOES preparation

MO mature seeds were harvested in Dschang District, West Cameroon (Africa) by the Cooperative of Medical Plant Producers SOCOPOMO. MO seeds were sun-dried and stored until use. In our laboratory, MOES from MO seed powder was prepared as previously described^31^.

### Plant microvesicles purification

MOES MVs were isolated from known concentrations of MOES, expressed as fresh plant weight (FW) equivalent per ml of distilled water. The aqueous extract was first filtered by a 0.45-µm pore filter, followed by a 0.22 µm pore filter, and centrifuged at 13,000 × *g* for 5 minutes. MOES MVs were stored at +4 °C until use.

### Plant MVs characterization and quantification

The MOES MVs were quantified using a Megamix-Plus SSC standard microparticle kit for ILV detection (Biocytex, FRANCE). The Megamix-Plus SSC kit contained nanosized FITC-A-conjugated standardized particles of different sizes (100–160 nm, 160–200 nm, 200–240 nm, 240–500 nm). Gates for data acquisition of all vesicle samples were set according to Megamix-Plus SSC nanoparticle standard protocols. The concentration of MOES MVs (number of events/µl) was calculated using CytoFLEX (Beckman Coulter, USA), and 150,000 events were acquired in the 100–500 nm range. CytExpert 2.2 software (Beckman Coulter, USA) was used for MOES MVs quantification: in three independent measurements for each of three different MOES MVs purifications, the number of MOES MVs contained in 1 mg (FW) of MOES was 16921 ± 617.

### Protein, RNA, DNA, and lipid detection in MOES MVs

For protein extraction, MOES MVs were lysed in RIPA buffer (50 mm Tris-HCl, pH 8.0, 150 mm NaCl, 12 mm deoxycholic acid, 0.5% Nonidet P-40, and protease and phosphatase inhibitors), and proteins were quantified using the Bradford method.

Total RNA was extracted from MOES MVs using TRIzol (Life Technologies | Thermo Fisher Scientific–US) according to the manufacturer’s protocol.

DNA was extracted from MOES MVs using a NucleoSpin DNA kit (MACHEREY-NAGEL, Germany).

The content of total RNA, DNA, and protein was measured using a NanoDrop Light Spectrophotometer (Thermo Fisher Scientific, USA). In addition to the spectrophotometer measurement method, flow cytometry was used to analyze the presence of RNA, DNA and lipids in MOES MVs using specific probes. For RNA detection, the MOES MVs were labeled with Invitrogen Molecular Probes SYTO RNA Select Green Fluorescent Cell Stain (SYTO RNA) (Thermo Fisher, USA) in accordance with the manufacturer’s instructions. The DNA content in MOES MVs was measured using the propidium iodide (PI) method, as described in the Materials and Methods in the Cell Cycle and Apoptosis assays section. For lipid detection, the MOES MVs were stained with BODIPY fluorescent probe (Thermo Fisher Scientific, USA) and incubated at +4 °C for 30 minutes.

Stained cells were analyzed using a CytoFLEX flow cytometer (Beckman Coulter, USA) and CytExpert 2.2 software (Beckman Coulter, USA).

The acquisition gate was set according to Megamix-Plus SSC nanoparticle standard protocols as previously described^20^ and reported in Supplementary data.

### MOES MVs delivery

Peripheral blood mononuclear cells (PBMCs) from healthy donors (HDs) and Jurkat and HeLa cell lines were treated with MOES MVs and stained with SYTO RNA, as described above. After 30 minutes of treatment, PBMCs and Jurkat cells were analyzed using CytoFLEX (Beckman Coulter, USA). The HeLa cell line was observed under a LEICA DMI6000B inverted microscope and analyzed using ImageJ software analysis.

### *M. oleifera* small RNA pool extraction and characterization

The *mol*-sR pool was extracted from MOES and MOES MVs by a NucleoSpin miRNA kit (MACHEREY-NAGEL, Germany). The *mol-*sR content was determined using a NanoDrop Light Spectrophotometer (Thermo Fisher Scientific, USA), and the *mol-*miRs contained in the *mol*-sR pool were characterized through quantitative RT-PCR. The presence and concentration of the most conserved *mol*-miRs were validated and measured as previously reported^16,31,58^. In detail, a Bio-Rad thermal cycler (IQ5) was used, with the amplification parameters set according to the instructions the EXIQON predesigned primers (Table S1). The relative expression of *mol*-miRs was quantified by the 2^−ΔΔCt^ method, where 5S RNA was applied as an internal control.

### Cell line culture

Human Jurkat E6-1 lymphoid cells mycoplasma free (American Type Culture Collection, USA) were grown in suspension culture at a density of 0.7 × 10^6^ cells/ml in Roswell Park Memorial Institute (RPMI) 1640 medium (Invitrogen, USA).

The HeLa human cervix epithelioid carcinoma cell line, mycoplasma free (American Type Culture Collection, USA) was grown as an adherent monolayer culture at a density of 0.15 × 10^6^ cells/ml in Dulbecco’s Modified Eagle Medium (Invitrogen, USA). The media of both culture types were supplemented with 10% fetal bovine serum (fetal bovine serum, Invitrogen, Germany), 2 mm glutamine (HyClone, UK), 50 U/ml penicillin and 50 U/ml streptomycin (HyClone, UK).

Human PBMCs from HDs were obtained from 17 individuals attending the local blood transfusion unit of Policlinico ‘Tor Vergata’ in Rome^31^. PBMCs were separated as previously described^59^. Cell lines and PBMCs were cultured at 37 °C in a 5% CO_2_ humidified atmosphere in the presence or absence of treatment.

### Treatment with MOES and MOES MVs

For the assessment of the cytotoxic effects of MOES and MOES MVs, 0.15 × 10^6^ Jurkat cells/ml, 0.5 × 10^5^ HeLa cells/ml and 1 × 10^6^ PBMCs/ml from HDs were treated for 72 h with MOES at concentrations ranging from 1 mg to 50 mg/ml of culture medium or MOES MVs extracted from the same concentrations of MOES (number of MVs present in mg/ml of MOES). PBMCs were co-stimulated with 20 U/ml IL2 (Merck KGaD, Germany).

For proliferation and apoptosis studies, Jurkat cells were placed in flasks and treated with 1 mg/ml MOES or MOES MVs purified from 10 mg MOES for 72 h.

HeLa cells were treated with 5 mg/ml MOES or MOES MVs purified from 20 mg MOES for 72 h. For the analysis, cells were washed with PBS, harvested, and centrifuged, and the pellets were stored at −20 °C.

### Transfection of the *mol-*sR pool

Jurkat and HeLa tumor cells were transfected with *mol-*sRs at concentrations of 1 μg/ml and 5 μg/ml, respectively, for 72 h. Transfection was performed according to the lipofectamine method (Hi-Fect, Qiagen, HF) as previously described^16^.

### Cell death/viability assays

Cell viability and mortality rates were assessed by a 10% trypan blue (EuroClone S.p.A., Italy) exclusion test after 72 h of treatment.

### Calculation of EC50 and LD50

Cumulative results from at least three different measurements of three independent experiments performed were used to calculate the concentration of MOES and MOES MVs required to reduce cell viability by 50% (EC50) or to induce death in 50% of cells (LD50). EC50 and LD50 were evaluated in all cell lines by sigmoidal dose–response regression curves using Graph Pad Prism software as previously described^60^.

### Cell cycle and apoptosis assays

Apoptosis was assessed through flow cytometry analysis of isolated nuclei stained with PI (Sigma) using a CytoFLEX flow cytometer (Beckman Coulter, USA) as previously described^31,61,62^.

In brief, detectors and amplifier gains for forward and orthogonal scatter were adequately selected in order to simultaneously detect nuclei from viable, apoptotic, and necrotic cells. Events were gated on forward versus orthogonal scatter in such a way that degraded DNA from cell debris or from doublets were excluded and nuclei from viable, apoptotic, and necrotic cells were assayed.

Data acquisition and analysis were performed on a minimum of 150,000 events for each sample using CytExpert 2.2 software (Beckman Coulter, USA).

### Annexin/PI assay

Early apoptotic events were detected through double staining of cells with fluorescent annexin V and PI solution. For this purpose, the Annexin V-FITC apoptosis detection kit (BD Biosciences Pharmingen, San José, CA) was used according to the manufacturer’s instructions as previously described^63^.

Cells were analyzed immediately after staining by flow cytometry analysis using a CytoFLEX flow cytometer and CytExpert 2.2 software.

### Intracellular BCL2 staining

Intracellular B-cell lymphoma 2 (BCL2) expression was evaluated by flow cytometry analysis. After 72 h, untreated and treated cells were harvested, fixed and permeabilized with 70% ethanol and incubated with PE-conjugated anti-human BCL2 (BD Biosciences, USA). Stained cells were analyzed using CytoFLEX (Beckman Coulter, USA) and CytExpert 2.2 software (Beckman Coulter, USA).

### Mitochondrial activity assays

#### JC-1 assay

To assess the mitochondrial membrane potential in both tumor cell lines, an assay was performed using the MitoProbe JC-1 assay kit for flow cytometry (Molecular Probes Europe BV, Leiden, The Netherlands), as previously described^36,53^. Mitochondrial depolarization is indicated by a decrease in the red/green fluorescence intensity ratio. Cells were analyzed using CytoFLEX (Beckman Coulter, USA) and CytExpert 2.2 software, counting a total of 100,00 events.

#### Mito Tracker Red FM

Jurkat and HeLa cells treated with MOES, MOES MVs and the *mol*-sR pool were stained with Mito Tracker Red FM probe according to the manufacturer’s instructions (Thermo Fisher, USA). Red was detected by FL-3 filters. The analysis was assessed using CytoFLEX (Beckman Coulter, USA) and CytExpert 2.2 software, counting a total of 100,00 events in live cells gates.

### Bioinformatics analysis

The *mol*-miR-human mRNA interactions were studied using a bioinformatics approach as previously described^16,20,29^. Specifically, we investigated the *mol*-miR-human mRNA interactions of genes involved in apoptosis.

### Statistical analysis

All data are presented as the mean ± standard deviation (SD) of at least three independent experiments in triplicate performed on HeLa cells, Jurkat cells and on PBMCs from HDs (*n* = 17). Data analyses were performed using the SPSS statistical software system (version 17.0 for Windows, USA).

Comparisons between treated and untreated cells for the results on the trypan blue assay, apoptosis assay, and BCL2 intracellular protein expression were all conducted using *t* test. Trypan blue assay results from the different concentrations of extracts and Annexin V assay were analyzed using analysis of variance (ANOVA) and a Bonferroni significant difference test as a multiple comparison test. Significant differences are shown as *p < 0.05, **p < 0.01, and ***p < 0.001. For non-parametric correlations, a Pearson correlation coefficient was calculated.

## Supplementary information

supplementary figures and tables legends

Figure S1

Figure S2

Table S1

Table S2
