# Supplementary material for: Effect of microvesicles from Moringa oleifera containing miRNA on proliferation and apoptosis in tumor cell lines
Source: Cell Death Discov. 2020 Jun 4;6:43. doi: 10.1038/s41420-020-0271-6 (PMC7272625; doi:10.1038/s41420-020-0271-6)
Supplement: Supplementary file 1 — supplementary figures and tables legends [file 41420_2020_271_MOESM1_ESM.docx]

Potestà et al. Supplementary Figures and Tables Ledends

**Supplementary Figure S1:** **Gating strategy.** Considering the MVs gate made respect to SSC-H and FSC-H parameters on the reference beads (A left panel), the sample of MOES MVs has been evaluated (A right panel).

50 microlitres of sample were recorded, in order to have an evaluation of the MVs content in the sample.

Five gates were designed for each size range of control beads population (beads below 100 nm, between 100 and 150, 160.200, 200-240 and 240-500 nm).

The analysis of MOES MVs using this gating strategy showed that the greatest number of MVs had a size between 240 and 500 nm.

Analyzing the MOES MVs present in the total MVs gate, the presence of RNA, lipids, and DNA has been assessed.

The MOES MVs were marked as follows: SYTO RNA 488 (B top panel), BODIPY-FL 488 (B center panel), and Propidium iodide (B foot panel). In this figure pseudo dot plots of unmarked MVs sample (-; left panels) and stained samples (+; right panels) are represented.

The quadrants allow to evaluate the positivity in the stained samples, with the different probes: MVs positive for the probes are those present in the Low Right quadrant (LR).

**Figure S2. Strategies for the analysis of apoptosis and cell cycle by Flow cytometry.**

For the cell population (panel A, Cells red gate) has been set up a dot plot Annexin V *vs* Propidium Iodide (panel B) and the quadrants were placed on an unmarked sample.

The same quadrants were used for the evaluation of the cells marked with Annexin V and Propidium Iodide, of which a representative experiment is presented in Figure 3.

For the analysis of the cycle and apoptosis, the cells were stained with PI. In the cell gate (panel C), 20000 events were recorded for each sample. The cycle analysis has been reported on a histogram plot on the FL-2-A (panel D). The different phases of the cell cycle were evaluated both as number and percentage (%) of PI positive events in untreated and treated Jurkat.

Representative histograms overlay of the cell cycle of untreated cells (red line), and treated cells (black line) (MOES *vs* untreated panel E and MOES MVs *vs* untreated panel F).

**Table S1.** Sequences of miRNA primers for *Moringa oleifera* miR qPCR analysis

**Table S2**: Cell Cycle analysis of Jurkat and HeLa cells treated with MOES and MOES MVs after 48 hours of incubation.
