# Supplementary figures and images for: Effect of microvesicles from Moringa oleifera containing miRNA on proliferation and apoptosis in tumor cell lines
Source: Cell Death Discov. 2020 Jun 4;6:43. doi: 10.1038/s41420-020-0271-6 (PMC7272625; doi:10.1038/s41420-020-0271-6)

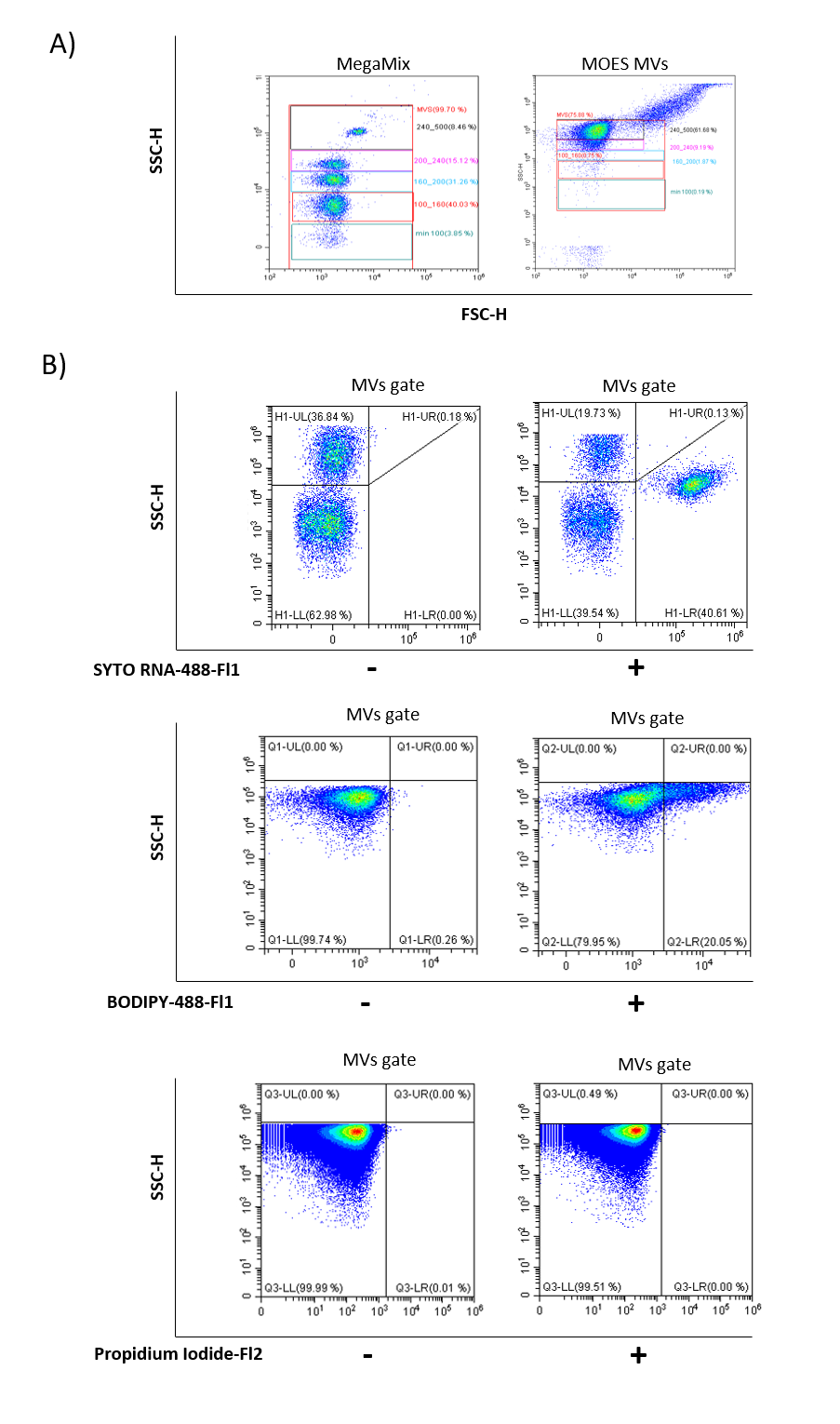

Supplement: Supplementary file 2 — Figure S1 [file 41420_2020_271_MOESM2_ESM.tif]

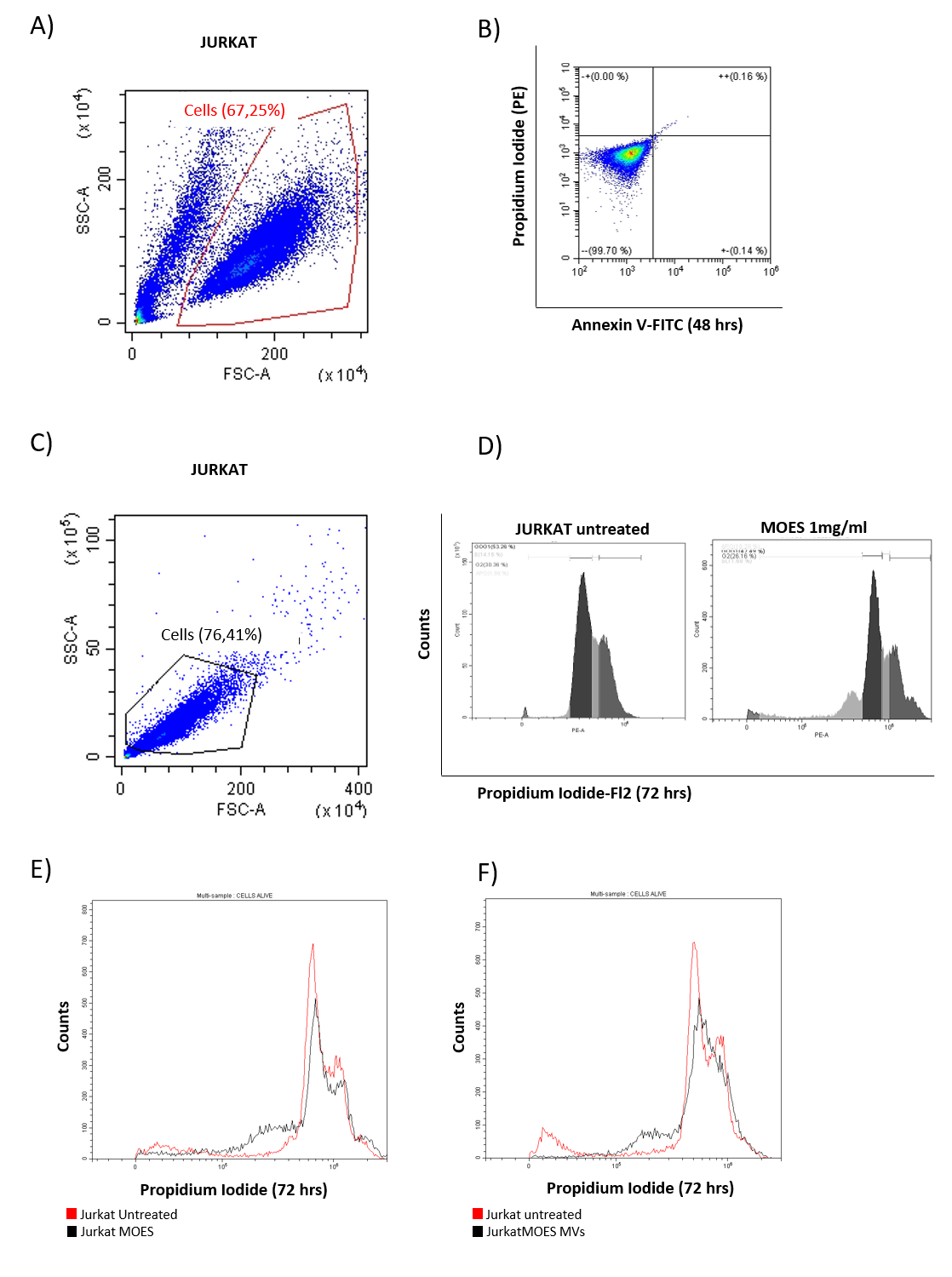

Supplement: Supplementary file 3 — Figure S2 [file 41420_2020_271_MOESM3_ESM.tif]
