# Supplementary material for: Effect of microvesicles from Moringa oleifera containing miRNA on proliferation and apoptosis in tumor cell lines
Source: Cell Death Discov. 2020 Jun 4;6:43. doi: 10.1038/s41420-020-0271-6 (PMC7272625; doi:10.1038/s41420-020-0271-6)
Supplement: Supplementary file 4 — Table S1 [file 41420_2020_271_MOESM4_ESM.docx]

| **Table S1.** Sequences of miRNA primers for *Moringa oleifera* miR qPCR analysis | |
| --- | --- |
| ***mol* miRs** | **5’ - 3’ sequences** |
| miR-156a | CUGACAGAAGAGAGUGAGCAC |
| miR-159a | UUUGGAUUGAAGGGAGCUCUA |
| miR-159c | UUUGGAUUGAAGGGAGCUCCU |
| miR-160h | UGCCUGGCUCCCUGUAUGCCAUU |
| miR-162a | UCGAUAAACCUCUGCAUCCA |
| miR-166i | UCGGACCAGGCUUCAUUCCCCC |
| miR-167-5p | UGAAGCUGCCAGCAUGAUCUU |
| miR-168a | UCGCUUGGUGCAGGUCGGGAA |
| miR-171d | UGAUUGAGCCGUGCCAAUAU |
| miR-393a | CAUCCAAAGGGAUCGCAUUGA |
| miR-395a | CUGAAGUGUUUGGGGGAACUC |
| miR-396a | UUCCACAGCUUUCUUGAACAG |
| miR-396c | UUCCACAGCUUUCUUGAACGU |
| miR-397-5p | UCAUUGAGUGCAGCGUUGAUG |
| miR-398b | GGGUUGAUUUGAGAACAUAUG |
| miR-482b | UCUUUCCUAUCCCUCCCAUUCC |
| miR-858a | UUCGUUGUCUGUUCGACCUUG |
| miR-858b | UUCGUUGUCUGUUCGACCUUG |
| miR-2118a | CUACCGAUGCCACUAAGUCCCA |
| miR-20 | UGGUGGUGGUGGUGGUGGUGACA |
| miR-34 | UGGAGGUGGAGGUGGCGGUGG |
| 5S rRNA | CAGAGTCCTATGGCCGTGGAT |
