# Supplementary material for: Effect of microvesicles from Moringa oleifera containing miRNA on proliferation and apoptosis in tumor cell lines
Source: Cell Death Discov. 2020 Jun 4;6:43. doi: 10.1038/s41420-020-0271-6 (PMC7272625; doi:10.1038/s41420-020-0271-6)
Supplement: Supplementary file 5 — Table S2 [file 41420_2020_271_MOESM5_ESM.docx]

|  |  |  |  |  |  |
| --- | --- | --- | --- | --- | --- |
| Table S2: Cell Cycle analysis of Jurkat and HeLa cells treated with MOES and MOES MVs after 48 hours of incubation. | | | | | |
| Jurkat | events |  |  |  |  |
| 72h | live cells | G0/G1 | S | G2 | Sub- G1 |
| untreated | 20000 | 3001±201 | 4089±365 | 11371±236 | 1487±321 |
| MVs 10 mg/ml | 19869 | 4219±101 | 3497±171 | 7334±145* | 5008±124** |
| MOES 1mg/ml | 19985 | 3097±65 | 3568±451 | 5895±569* | 6589±69** |
|  |  |  |  |  |  |
| HeLa | events |  |  |  |  |
| 72h | live cells | G0/G1 | S | G2 | Sub- G1 |
| untreated | 20000 | 5000±147 | 5014±547 | 9846±114 | 564±32 |
| MVs 20 mg/ml | 19869 | 4598±251 | 4564±741 | 7334±184* | 3897±125** |
| MOES 5 mg/ml | 19985 | 4561±354 | 3598±323 | 6874±252* | 5591±471** |
|  |  |  |  |  |  |
| Jurkat | % |  |  |  |  |
| 72h | live cells | G0/G1 | S | G2 | Sub-G1 |
| untreated | 78,00±2,63 | 17,86±1,54 | 17,95±1,45 | 59,31±4,45 | 5,26±1,45 |
| MVs 10 mg/ml | 74,56±5,23 | 19,65±2,45 | 15,57±1,78 | 48,56±2,45* | 15,56±2,45** |
| MOES 1mg/ml | 76,32±3,45 | 18,31±3,45 | 16,54±3,45 | 47,62±1,98* | 17,54±2,87** |
|  |  |  |  |  |  |
| HeLa | % |  |  |  |  |
| 72h | live cells | G0/G1 | S | G2 | Sub-G1 |
| untreated | 69,12±3,5 | 20,29±1,45 | 15,48±4,54 | 57,96±5,78 | 7,4±1,54 |
| MVs 20 mg/ml | 68,45±4,56 | 17,28±2,15 | 21,21±3,45 | 48,32±1,5 | 14,65±1,89 |
| MOES 5 mg/ml | 69,15±5,45 | 18,56±2,54 | 19,21±3,12 | 45,56±2,65 | 17,54±2,54 |
| The analysis was carried out on a gate of alive cells. through the CytExpert software: 20000 events in Alive cells gate were recorded for each sample and through the cell cycle analysis the events for each phase of the cycle were estimated. The supplementary Figure S2 show the gating strategies used.  Mean±SD of six independent measurements, * p<0,05, ** p<0,01; MOES or MOES MVs *vs* untreated cells (ANOVA and a Bonferroni multiple comparison test were used). | | | | | |
